# Identification of prognostic factors for predicting survival of patients with malignant adrenal tumors: A population-based study

**DOI:** 10.3389/fonc.2022.930473

**Published:** 2022-10-17

**Authors:** Keyi Wang, Tao Zhang, Jinliang Ni, Jianghong Chen, Houliang Zhang, Guangchun Wang, Yongzhe Gu, Bo Peng, Weipu Mao, Jianping Wu

**Affiliations:** ^1^ Department of Urology, Putuo People's Hospital, Tongji University School of Medicine, Shanghai, China; ^2^ Department of Urology, Shanghai Tenth People’s Hospital, School of Medicine, Tongji University, Shanghai, China; ^3^ Shanghai Clinical College, Anhui Medical University, Hefei, China; ^4^ Department of Surgery, Traditional Chinese Medicine Hospital of Jiulongpo District, Chongqing, China; ^5^ Department of Neurology, Shanghai Tenth People’s Hospital, School of Medicine, Tongji University, Shanghai, China; ^6^ Department of Urology, Affiliated Zhongda Hospital of Southeast University, Nanjing, China

**Keywords:** malignant adrenal tumors, prognostic factors, nomograms, SEER program, survival

## Abstract

**Background:**

This study aimed to identify the prognostic factors for overall survival (OS) and cancer-specific survival (CSS) in patients with malignant adrenal tumors and establish a predictive nomogram for patient survival.

**Methods:**

The clinical characteristics of patients diagnosed with malignant adrenal tumors between 1988 and 2015 were retrieved from the Surveillance, Epidemiology and End Results (SEER) database. As the external validation set, we included 110 real-world patients from our medical centers. Univariate and multivariate Cox regressions were implemented to determine the prognostic factors of patients. The results from Cox regression were applied to establish the nomogram.

**Results:**

A total of 2,206 eligible patients were included in our study. Patients were randomly assigned to the training set (1,544; 70%) and the validation set (662; 30%). It was determined that gender, age, marital status, histological type, tumor size, SEER stage, surgery, and chemotherapy were prognostic factors that affected patient survival. The OS prediction nomogram contained all the risk factors, while gender was excluded in the CSS prediction nomogram. The receiver operating characteristic (ROC) curve and decision curve analysis (DCA) indicated that the nomogram had a better predictive performance than SEER stage. Moreover, the clinical impact curve (CIC) showed that the nomograms functioned as effective predictive models in clinical application. The C-index of nomogram for OS and CSS prediction was 0.773 (95% confidence interval [CI]: 0.761–0.785) and 0.689 (95% CI: 0.675–0.703) in the training set. The calibration curves exhibited significant agreement between the nomogram and actual observation. Additionally, the results from the external validation set also presented that established nomograms functioned well in predicting the survival of patients with malignant adrenal tumors.

**Conclusions:**

The following clinical variables were identified as prognostic factors: age, marital status, histological type, tumor size, SEER stage, surgery, and chemotherapy. The nomogram for patients with malignant adrenal tumors contained the accurate predictive performance of OS and CSS, contributing to optimizing individualized clinical treatments.

## Introduction

Adrenal tumors are prevalent in urology. They may develop from the adrenal cortex or medulla, or they may be secondary lesions. Depending on the different subtypes, they are classified as benign or malignant (1). Malignant adrenal tumors mainly include pheochromocytoma or adrenal cortical carcinoma (ACC). Only a minority of adrenal tumors are diagnosed as pheochromocytoma or ACC with the characterization of distant metastasis and capsule infiltration (2).

Malignant adrenal tumors are aggressive and commonly result in a poor prognosis. The worldwide estimated incidence of adult ACC is between 0.5 and 2.0 per million people annually, and that of pheochromocytoma is 1 to 2 cases per million (2, 3). However, the mortality rate of ACC accounts for 0.02% to 0.2% of all cancer-related deaths, and the 5-year survival rate is less than 40% for pheochromocytoma patients (4). Surgical excision is the most effective therapy for malignant adrenal tumors. Other multimodal therapies are available for advanced malignant adrenal tumors but have several limitations and severe side effects (5). Accurate predictions for patient survival would contribute to the personalized therapy management, which prolongs survival time. Accordingly, timely distinguishing prognostic factors and establishing the prognostic model will function well in the related predictions. In recent years, the nomogram has been one of the most widely used statistical methods to predict tumor prognosis considering its unique calculation method (6). This study aimed to identify the prognostic factors and establish the nomogram predicting the prognosis of malignant adrenal tumor patients to aid clinical treatment.

In this study, we evaluated data from the Surveillance, Epidemiology and End Results (SEER) database, and further investigated the prognostic factors influencing the survival of patients with malignant adrenal tumors. Additionally, the final nomograms were developed based on the results of Cox regressions from the patient’s information in the SEER database. It is helpful to confirm the relationship between different clinical factors and patients’ overall survival (OS) and cancer-specific survival (CSS).

## Patients and methods

### Data source

**Figure 1:**
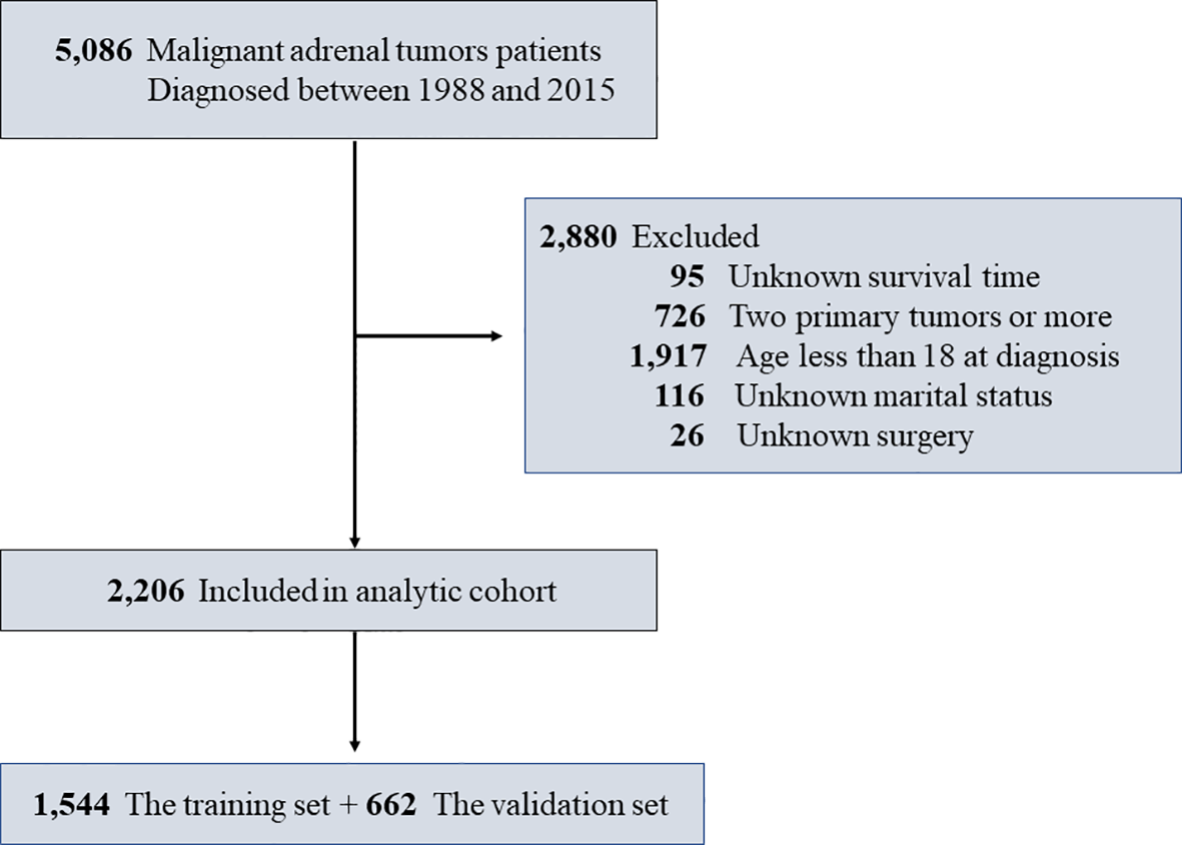
Study design flowchart of specific patient screening process.

The clinical data of patients diagnosed with malignant adrenal tumors from 1988 to 2015 were obtained from the SEER database through SEER*Stat software [version 8.3.5; SEER 18 Regs Custom Data (with additional treatment fields), Nov 2018 Sub (1975–2016 varying) database]. The SEER database, as one of the largest public cancer datasets, covered 28% of the U.S. population (6). Morphological ICD-O-3 nomenclature and Topographical ICD-O-3/WHO 2008 for SEER Site recode were applied in the determination of malignant adrenal tumors (7). The exclusion criteria adopted in our study were as follows: (a) unknown survival time; (b) two primary tumors or more; (c) age less than 18 at diagnosis; (d) unknown marital status; and (e) unknown surgery. The detailed exclusion protocol is shown in **Figure 1**. Because of the retrospective nature of this study, informed consent was not required. The Ethics Committee of Shanghai Tenth People’s Hospital, School of Medicine, Tongji University approved all procedures in this study.

### Study variables

The patients’ characteristics retrieved from the SEER database included gender, the age of diagnosis, race, marital status, tumor primary site, tumor laterality, histological type, tumor size, SEER stage, surgery, chemotherapy, and radiotherapy. The age of diagnosis was divided into four groups, namely, ≤40, 41–60, 61–80, and >80. Marital status contained four subgroups: married, divorced/separated, widowed, and single. As for the tumor primary site, there were three kinds, namely, the adrenal gland, NOS; cortex of the adrenal gland; and medulla of the adrenal gland. The histological type involved ACC and pheochromocytoma. The tumor laterality was classified into four kinds, namely, right, left, bilateral, and unknown. Additionally, the tumor size and SEER stage were also divided into subgroups. The tumor size had two subgroups in terms of diameter: ≤5 cm and >5 cm. SEER stage contained four subgroups: localized, regional, distant, and unstage. This study took OS and CSS as the endpoints.

### Statistical analysis

This study conducted univariate and multivariate Cox regression analyses for the determination of clinical risk factors. The univariate Cox regressions were applied to measure the single variable’s predictive performance towards patient survival, and the multivariate Cox regressions were conducted to determine the independent prognostic factors. The Cox analysis results were used to develop and validate nomograms for predicting the survival of patients with malignant adrenal tumors. Receiver operating characteristic (ROC) curves and decision curve analysis (DCA) were applied to evaluate the predictive performance of the nomogram and SEER stage. The clinical impact curve (CIC) was performed to measure the predictive value. The predictive accuracy of the nomogram was assessed by the concordance index (C-index) and a calibration curve. The C-index revealed the excellent predictive performance of the nomogram when the value was close to 1. The calibration curve falling on the 45° diagonal indicated the great predictive accuracy of the nomogram. All statistical analyses in this study were performed through Statis7tical Package for the Social Sciences software (version 20.0; SPSS Inc., Chicago, IL, USA). The “rms” and “rmda” R packages were conducted for the development of the nomogram through R software version 3.5.1 (http://www.R-project.org). The result was considered statistically significant in the statistical analysis with *p*-value <0.05 (two-sided).

## Results

### Clinical characteristics

A total of 2,206 eligible patients were enrolled in this study. The eligible patients were randomly assigned to the training set (*n* = 1,544; 70%) and the validation set (*n* = 662; 30%). Moreover, there were 110 eligible patients included in the external validation set with the clinical features collected from our medical centers consisting of People’s Hospital of Putuo District, Shanghai Tenth People’s Hospital, and Affiliated Zhongda Hospital of Southeast University from 2016 to 2020. In terms of gender, there were more female patients (*n* = 1,223; 55.4%) than male patients. There were 878 (39.8%) patients aged between 41 and 60, considered as the majority. As regards race, most of the patients were white (*n* = 1,804; 81.8%). The patients’ marital status was mainly married (*n* = 1,290; 58.5%). For the tumor primary site, the three subgroups were 1,333 (60.4%; adrenal gland, NOS), 748 (33.9%; cortex of the adrenal gland), and 125 (5.7%; medulla of the adrenal gland). The most prevalent laterality of the tumor was left (*n* = 1,083; 49.1%). As for the tumor histological type, most of the patients had ACC (*n* = 1,322; 59.9%). The tumor size was mainly concentrated on the >5 group (*n* = 1,499; 68.0%). The distant group of SEER stage contained 936 patients (42.4%), the most among the four subgroups. Patients with malignant adrenal tumors were mainly treated with surgery (*n* = 1,411; 64.0%), while radiotherapy (*n* = 275; 12.5%) and chemotherapy (*n* = 651; 29.5%) were rarely used. The baseline characteristics of patients are detailed in **Table 1**.

**Table 1:** Baseline demographic and clinical characteristics.

Variables	All patients *n* (%)	Training set	Validation set
		*n* (%)	*n* (%)
Total	2,206	1,544 (70.0)	662 (30.0)
Gender			
Male	983 (44.6)	694 (44.9)	289 (43.7)
Female	1,223 (55.4)	850 (55.1)	373 (56.3)
Age, years			
≤40	417 (18.9)	280 (18.1)	137 (20.7)
41–60	878 (39.8)	632 (40.9)	246 (37.2)
61–80	752 (34.1)	518 (33.5)	234 (35.3)
>80	159 (7.2)	114 (7.4)	45 (6.8)
Race			
White	1,804 (81.8)	1,268 (82.1)	536 (81.0)
Black	216 (9.8)	148 (9.6)	68 (10.3)
Others	186 (8.4)	128 (8.3)	58 (8.8)
Marital status			
Married	1,290 (58.5)	895 (58.0)	395 (59.7)
Divorced/Separated	216 (9.8)	143 (9.3)	73 (11.0)
Widowed	213 (9.7)	155 (10.0)	58 (8.8)
Single	487 (22.1)	351 (22.7)	136 (20.5)
Primary site			
Adrenal gland, NOS	1,333 (60.4)	926 (60.0)	407 (61.5)
Cortex of adrenal gland	748 (33.9)	529 (34.3)	219 (33.1)
Medulla of adrenal gland	125 (5.7)	89 (5.8)	36 (5.4)
Laterality			
Right	935 (42.4)	657 (42.7)	278 (42.0)
Left	1,083 (49.1)	756 (49.0)	327 (49.4)
Bilateral	28 (1.3)	17 (1.1)	11 (1.7)
Unknown	160 (7.3)	114 (7.4)	46 (6.9)
Histological type			
Adrenal cortical carcinoma	1,322 (59.9)	927 (60.0)	395 (59.7)
Pheochromocytoma	294 (13.3)	204 (13.2)	90 (13.6)
Unknown	590 (26.7)	413 (26.7)	177 (26.7)
Tumor size, cm			
≤5	237 (10.7)	155 (10.0)	82 (12.4)
>5	1,499 (68.0)	1,050 (68.0)	449 (67.8)
Unknown	470 (21.3)	339 (22.0)	131 (19.8)
SEER stage			
Localized	697 (31.6)	485 (31.4)	212 (32.0)
Regional	380 (17.2)	261 (16.9)	119 (18.0)
Distant	936 (42.4)	663 (42.9)	273 (41.2)
Unstage	193 (8.7)	135 (8.7)	58 (8.8)
Surgery			
No	795 (36.0)	566 (36.7)	229 (34.6)
Yes	1,411 (64.0)	978 (63.3)	433 (65.4)
Chemotherapy			
No/Unknown	1,931 (87.5)	1,352 (87.6)	579 (87.5)
Yes	275 (12.5)	192 (12.4)	83 (12.5)
Radiotherapy			
No/Unknown	1,555 (70.5)	1,101 (71.3)	454 (68.6)
Yes	651 (29.5)	443 (28.7)	208 (31.4)

SEER, Surveillance, Epidemiology, and End Results.

### Evaluation of prognostic factors by regression analysis

Univariate and multivariate Cox regressions were implemented in this work for determining risk factors for OS and CSS of patients in the training set. The included clinical variables in the analyses consisted of gender, the age of diagnosis, race, marital status, tumor primary site, tumor laterality, histological type, tumor size, SEER stage, surgery, chemotherapy, and radiotherapy. For the analyses of OS, the results are detailed in **Table 2**, and the results of risk factors for CSS are shown in **Table 3**.

**Table 2:** Univariate and multivariate analysis of overall survival (OS) rates in the training set.

Variables	No. of patients	Univariate analysis	Multivariate analysis^a^	
		p-value	HR (95% CI)	p-value
Gender		0.008		
Male	694		Reference	
Female	850		0.83 (0.74–0.94)	0.003
Age, years		<0.001		
≤40	280		Reference	
41–60	632		1.14 (0.96–1.36)	0.148
61–80	518		1.69 (1.40–2.03)	<0.001
>80	114		2.79 (2.10–3.70)	<0.001
Race		0.583		
White	1,268			
Black	148			
Others	128			
Marital status		<0.001		
Married	895		Reference	
Divorced/Separated	143		1.26 (1.03–1.55)	0.025
Widowed	155		1.35 (1.10–1.66)	0.004
Single	351		1.08 (0.93–1.26)	0.302
Primary site		<0.001		
Adrenal gland, NOS	926		Reference	
Cortex of adrenal gland	529		–	0.240
Medulla of adrenal gland	89		–	0.620
Laterality		<0.001		
Right	657		Reference	
Left	756		–	0.398
Bilateral	17		–	0.218
Unknown	114		–	0.073
Histological type		<0.001		
Adrenal cortical carcinoma	927		Reference	
Pheochromocytoma	204		0.46 (0.37–0.56)	<0.001
Unknown	413		1.10 (0.96–1.27)	0.185
Tumor size, cm		<0.001		
≤5	155		Reference	
>5	1,050		1.44 (1.14–1.81)	0.002
Unknown	339		1.11 (0.86–1.42)	0.424
SEER stage		<0.001		
Localized	485		Reference	
Regional	261		1.63 (1.34–1.97)	<0.001
Distant	663		3.75 (3.14–4.46)	<0.001
Unstage	135		1.74 (1.34–2.24)	<0.001
Surgery		<0.001		
No	566		Reference	
Yes	978		0.43 (0.37–0.50)	<0.001
Chemotherapy		<0.001		
No/Unknown	1,352		Reference	
Yes	192		0.85 (0.74–0.98)	0.029
Radiotherapy		0.032		
No/Unknown	1,101		Reference	
Yes	443		–	0.311

OS, overall survival; HR, hazard ratio; CI, confidence interval; SEER, Surveillance, Epidemiology, and End Results.

a Model was adjusted by gender, age, marital status, primary site, laterality, histological type, tumor size, SEER stage, surgery, and chemotherapy.

**Table 3:** Univariate and multivariate analysis of cancer-specific survival (CSS) rates in the training set.

Variables	No. of patients	Univariate analysis	Multivariate analysis^a^	
		p-value	HR (95% CI)	p-value
Gender		0.256		
Male	694			
Female	850			
Age, years		<0.001		
≤40	280		Reference	
41–60	632		1.12 (0.95–1.33)	0.186
61–80	518		1.67 (1.40–2.00)	<0.001
>80	114		2.98 (2.30–3.87)	<0.001
Race		0.146		
White	1,268			
Black	148			
Others	128			
Marital status		0.016		
Married	895		Reference	
Divorced/Separated	143		–	0.120
Widowed	155		–	0.053
Single	351		–	0.646
Primary site		<0.001		
Adrenal gland, NOS	926		Reference	
Cortex of adrenal gland	529		–	0.322
Medulla of adrenal gland	89		–	0.654
Laterality		<0.001		
Right	657		Reference	
Left	756		–	0.361
Bilateral	17		–	0.263
Unknown	114		–	0.057
Histological type		<0.001		
Adrenal cortical carcinoma	927		Reference	
Pheochromocytoma	204		0.47 (0.39–0.58)	<0.001
Unknown	413		1.13 (0.98–1.30)	0.090
Tumor size, cm		<0.001		
≤5	155		Reference	
>5	1,050		1.44 (1.14–1.81)	0.002
Unknown	339		1.12 (0.87–1.43)	0.373
SEER stage		<0.001		
Localized	485		Reference	
Regional	261		1.64 (1.35–1.98)	<0.001
Distant	663		3.75 (3.15–4.47)	<0.001
Unstage	135		1.76 (1.36–2.27)	<0.001
Surgery		<0.001		
No	566		Reference	
Yes	978		0.44 (0.37–0.51)	<0.001
Chemotherapy		<0.001		
No/Unknown	1,352		Reference	
Yes	192		0.85 (0.74–0.97)	0.020
Radiotherapy		0.028		
No/Unknown	1,101		Reference	
Yes	443		–	0.406

CSS, cancer-specific survival; HR, hazard ratio; CI, confidence interval; SEER, Surveillance, Epidemiology, and End Results.

a Model was adjusted by age, marital status, primary site, laterality, histological type, tumor size, SEER stage, surgery, and chemotherapy.

The prognostic factors for OS were gender, the age of diagnosis, marital status, histological type, tumor size, SEER stage, surgery, chemotherapy, and radiotherapy based on the results of univariate and multivariate Cox regression analyses. The univariate Cox regression results indicated that tumor primary site (*p* < 0.001) and tumor laterality (*p* < 0.001) were risk factors, which was inconsistent with the results from multivariate Cox regression. For the analyses of CSS, the risk factors excluded race due to the non-significant differences in statistical analysis (*p* = 0.146).

### Construction and verification of nomograms

**Figure 2:**
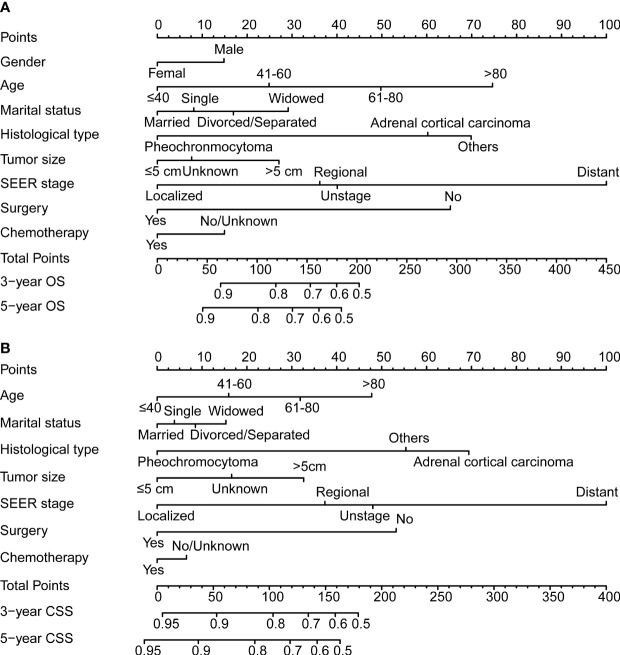
The 3- and 5-year nomogram model for overall survival (OS; **A**) and cancer-specific survival (CSS; **B**) for patients with malignant adrenal tumors.

The variables selected in the construction and verification of nomograms were screened according to the Cox regression results. The selected variables included gender, the age of diagnosis, marital status, histological type, tumor size, SEER stage, surgery, chemotherapy, and radiotherapy for the development of nomogram for OS, which excluded gender in the nomogram for CSS. The 3- and 5-year nomograms for predicting OS and CSS of patients with malignant adrenal tumors are exhibited in **Figures 2A, B**.

**Figure 3:**
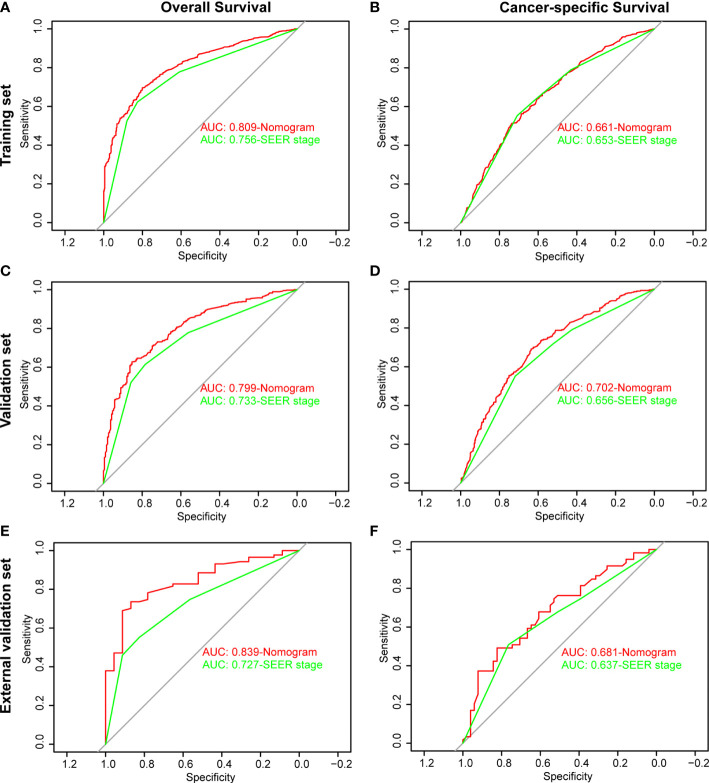
Receiver operating characteristic (ROC) analysis based on nomograms and SEER stage. **(A)** The ROC analysis of OS in the training set. **(B)** The ROC analysis of CSS in the training set. **(C)** The ROC analysis of OS in the validation set. **(D)** The ROC analysis of CSS in the validation set. **(E)** The ROC analysis of OS in the external validation set. **(F)** The ROC analysis of CSS in the external validation set.

**Figure 4:**
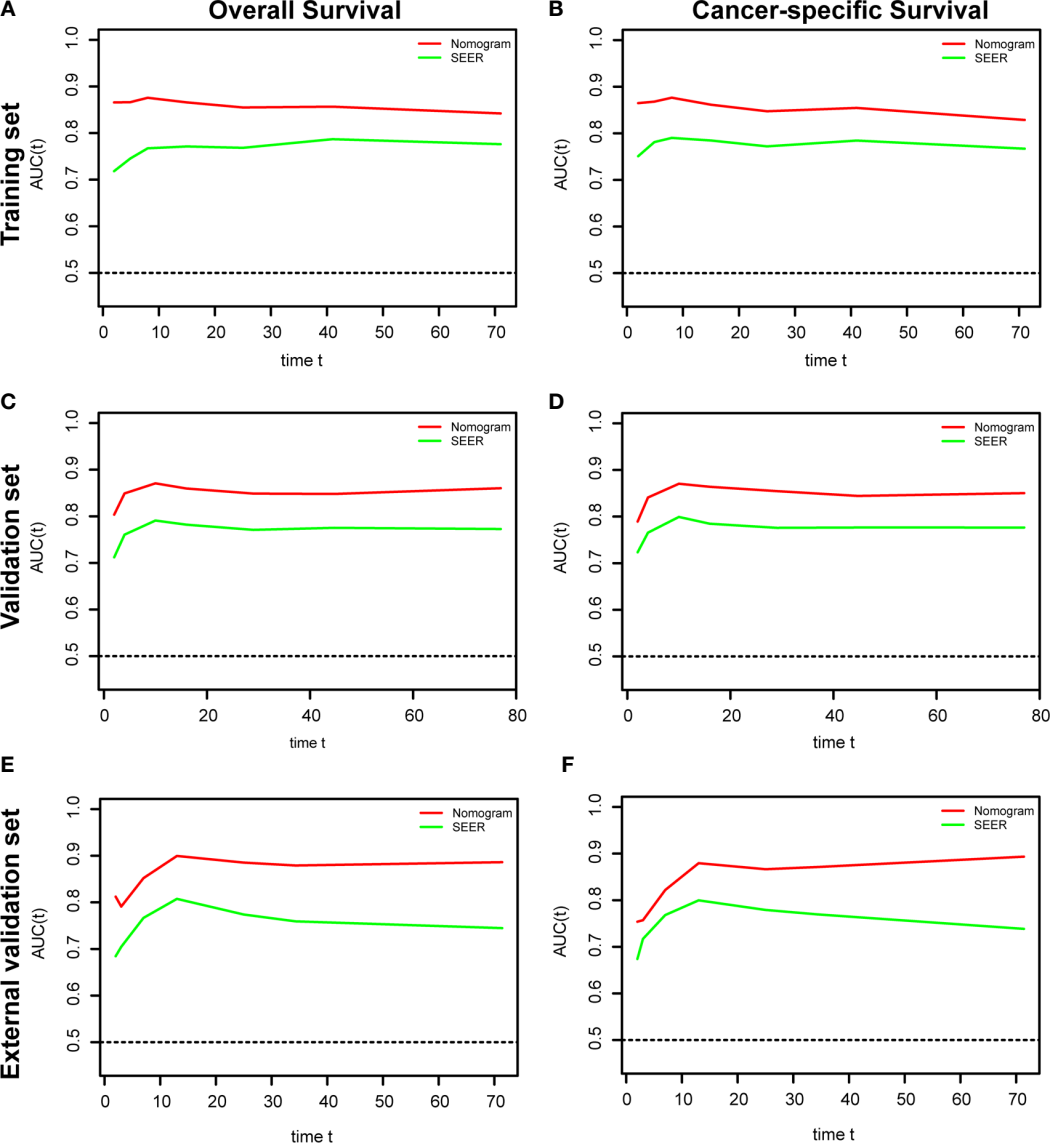
Time-dependent receiver operating characteristic (ROC) analysis based on nomograms and SEER stage. **(A)** The time dependent ROC analysis of OS in the training set. **(B)** The time-dependent ROC analysis of CSS in the training set. **(C)** The time-dependent ROC analysis of OS in the validation set. **(D)** The time-dependent ROC analysis of CSS in the validation set. **(E)** The time-dependent ROC analysis of OS in the external validation set. **(F)** The time-dependent ROC analysis of CSS in the external validation set.

**Figure 5:**
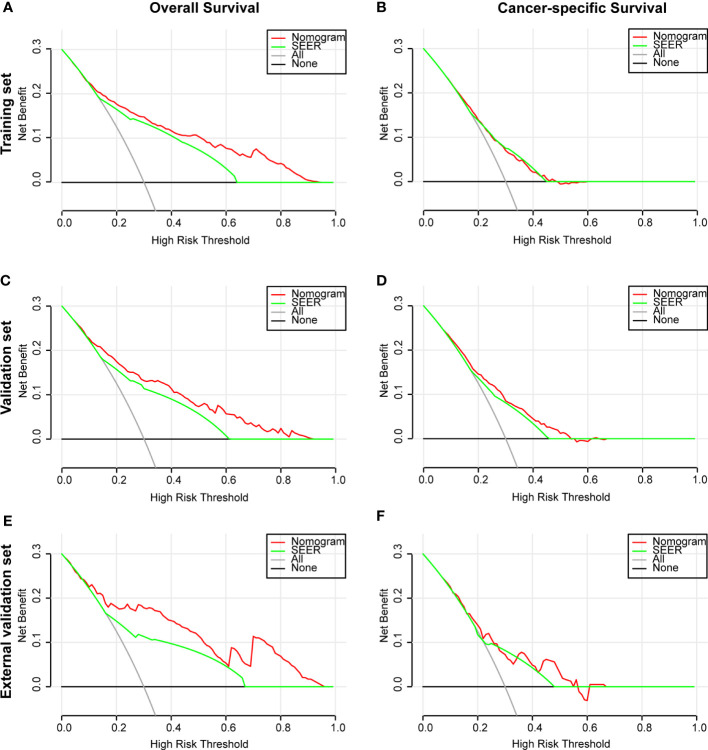
Decision curve analysis (DCA) based on nomograms and SEER stage. **(A)** DCA of OS for patients in the training set. **(B)** DCA of CSS for patients in the training set. **(C)** DCA of OS for patients in the validation set. **(D)** DCA of CSS for patients in the validation set. **(E)** DCA of OS for patients in the external validation set. **(F)** DCA of CSS for patients in the external validation set.

**Figure 6:**
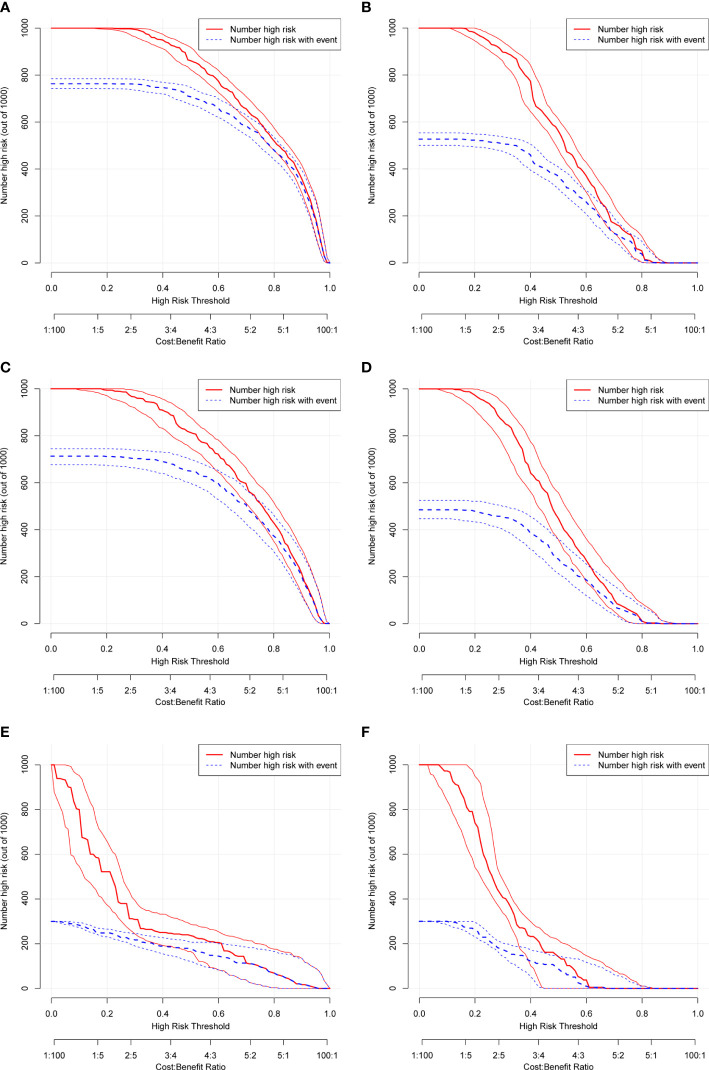
Clinical impact curve (CIC) based on nomograms. **(A)** CIC of OS for patients in the training set. **(B)** CIC of CSS for patients in the training set. **(C)** CIC of OS for patients in the validation set. **(D)** CIC of CSS for patients in the validation set. **(E)** CIC of OS for patients in the external validation set. **(F)** CIC of CSS for patients in the external validation set.

The ROC and DCA curves were applied in the evaluation of the nomogram and SEER stage. As shown in **Figure 3**, the nomogram for OS accompanied a better predictive performance than that of SEER stage in both the training set and the validation set (**Figures 3A, C**) with an area under the curve (AUC) of 0.809 (*p* < 0.001) and 0.799 (*p* < 0.001), respectively (**Table 4**). The ROC results for CSS prediction are shown in **Figures 3B, D**, and the nomogram for CSS also exhibited better performance compared with SEER stage for predicting prognosis in the validation set with an AUC of 0.702 (*p* = 0.001) (**Table 4**). The results from the external validation set also proved that the nomogram contained better predictive performances of OS (**Figure 3E**) and CSS (**Figure 3F**) than SEER stage. Additionally, the time-dependent ROC curves were established in this work, indicating the better predictive performances of nomograms than SEER stage (**Figure 4**). The results directly proved that nomograms functioned better in predicting OS (**Figures 4A, C**) and CSS (**Figures 4B, D**) in the two cohorts, and the results of the external validation set further proved the excellent predicting ability of nomograms (**Figures 4E, F**). The same conclusions were obtained from DCA curves (**Figure 5**). The nomogram for OS contained better clinical applicability than SEER stage in the training set and validation set (**Figures 5A, C**). For nomogram predicting CSS, the DCA curves indicated better performance in the validation set (**Figure 5D**), which was not obviously found in the training set (**Figure 5B**). As for the external validation set, **Figures 5E, F** show a better performance of nomograms in both OS and CSS. Meanwhile, the CIC curves also proved the outstanding predictive performances of the nomogram for OS in both the training set and the validation set (**Figures 6A, C**). The results of nomograms for CCS also exhibited a similar function in the clinical application (**Figures 6B, D**). The results from the external validation set also proved the above conclusion that nomograms contain better predictive performances (**Figures 6E, F**).

**Table 4:** Comparison of area under the curve (AUC) between the nomogram and SEER stages in patients with malignant adrenal tumors.

Characteristics		Training set			Validation set		
		AUC	95% CI	p-value	AUC	95% CI	p-value	
OS	Nomogram	0.809	0.789–0.829		0.799	0.767–0.829		
	SEER stage	0.756	0.733–0.777	<0.001	0.733	0.698–0.767	<0.001	
CSS	Nomogram	0.661	0.637–0.685		0.702	0.666–0.737		
	SEER stage	0.653	0.628–0.676	0.287	0.87656	0.619–0.692	0.001	

AUC, area under the curve; CI, confidence interval; SEER, Surveillance, Epidemiology, and End Results.

**Figure 7:**
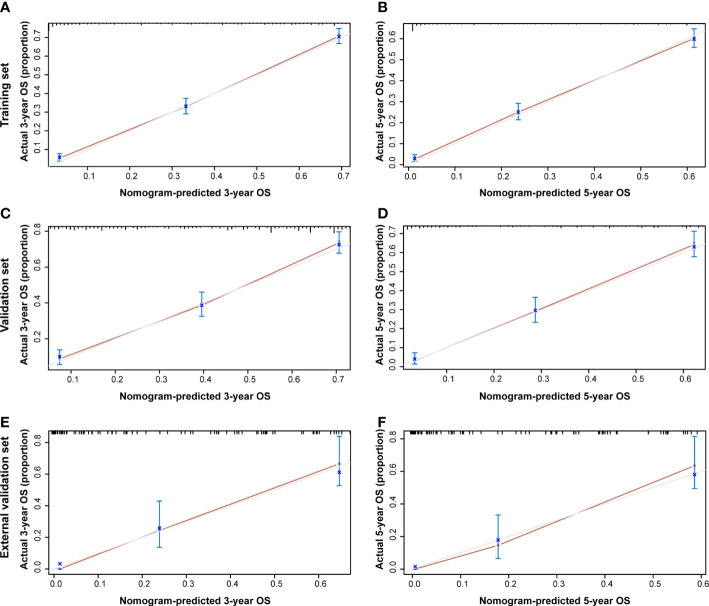
Calibration plot of the 3- and 5-year OS nomogram. The calibration curves of the 3-year **(A)** and 5-year **(B)** nomogram model for OS in the training set, respectively. The calibration curves of the 3-year **(C)** and 5-year **(D)** nomogram model for OS in the validation set. The calibration curves of the 3-year **(E)** and 5-year **(F)** nomogram model for OS in the external validation set.

**Figure 8:**
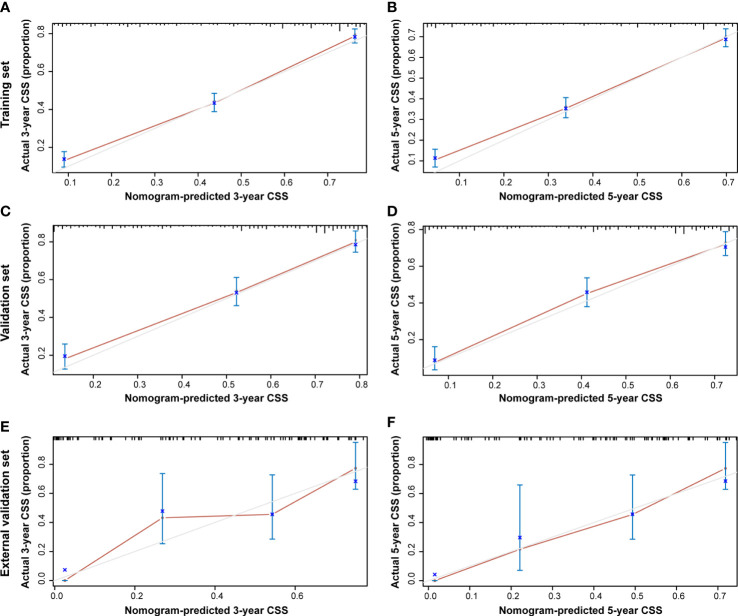
Calibration plot of the 3- and 5-year CSS nomogram. The calibration curves of the 3-year **(A)** and 5-year **(B)** nomogram model for CSS in the training set, respectively. The calibration curves of the 3-year **(C)** and 5-year **(D)** nomogram model for CSS in the validation set. The calibration curves of the 3-year **(E)** and 5-year **(F)** nomogram model for CSS in the external validation set.

Additionally, the C-index and calibration curve were conducted for the assessment of the nomogram in predicting accuracy. The calculated C-index of the nomogram for OS was 0.773 (95% CI: 0.761–0.785) and 0.762 (95% CI: 0.742–0.782) in the training set and validation set, respectively. As for the nomogram predicting CSS, the corresponding C-index in the two sets was 0.689 (95% CI: 0.675–0.703) and 0.692 (95% CI: 0.670–0.714). Simultaneously, the calibration curves showed the accuracy of the nomogram by fitting curves to 45° diagonal lines. As shown in **Figure 7**, the 3- and 5-year nomogram predicting OS exhibited an excellent agreement with the actual observations in the training set (**Figures 7A, B**), validation set (**Figures 7C, D**), and external validation set (**Figures 7E, F**). The 3- and 5-year nomogram calibration for CSS is shown in **Figure 8** (**Figures 8A, B** for the training set, **Figures 8C, D** for the validation set, and **Figures 8E, F** for the external validation set). The results of the C-index and calibration curve indicated the excellent accuracy of the nomogram in predicting prognosis.

## Discussion

In this study, we identified the prognostic factors, namely, age, marital status, histological type, tumor size, SEER stage, surgery, and chemotherapy. We then established the prognostic nomograms for OS and CSS in patients with malignant adrenal tumors. The nomograms could accurately predict the 3- and 5-year OS and CSS based on the Cox regression results. We included the above eight clinical variables to construct a nomogram to predict OS, while gender was excluded in the nomogram predicting CSS. The successful establishment of nomograms would contribute to optimizing personalized treatment and extending survival time in patients with malignant adrenal tumors.

The development of adrenal tumors is always accompanied by endocrine abnormalities. It is difficult to distinguish benign from malignant tumors in clinical settings (5). Malignant adrenal tumors mainly consist of two pathological types, ACC and pheochromocytoma (8). Both kinds of tumors have low morbidity, with nearly two new cases per 1 million people per year (8, 9). It was reported that the patients diagnosed with malignant adrenal tumors had metastases in different organs, such as the liver and lung (10, 11). Diagnosing malignant adrenal tumors requires a combination of clinical manifestations, imaging, and pathological results. Timely diagnosis and individualized treatment will prolong patients’ OS and CSS. Establishing an effective prognostic prediction model is necessary to assist clinicians in optimizing individualized treatments. Our work established nomograms in predicting the survival of patients, which assists in improving clinical treatment for the two kinds of malignant adrenal tumors.

The nomogram as a graphical tool functioned well in predicting prognosis because of its sample intuitive computing features (12, 13). It gave patients and clinicians a tangible interpretation of each predictive factor, which avoided complex input and calculation processes. A single clinical risk factor was examined for predicting OS and CSS, which were not involved in a comprehensive model. Meanwhile, the nomogram combined multiple clinical predictors to predict OS and CSS accurately. In this study, we concluded that gender, the age of diagnosis, marital status, histological type, tumor size, SEER stage, surgery, chemotherapy, and radiotherapy were the risk factors for patients with malignant adrenal tumors. We then developed the 3- and 5-year nomograms by including the variables affecting prognosis. The predictive performance of the nomograms was examined by ROC and DCA curves, which were compared with SEER stage simultaneously. Additionally, the CIC results also showed that those nomograms functioned well in clinical applications. The above results were further confirmed in the external validation set. It was considered the first attempt to establish a prognostic prediction model for patients with malignant adrenal tumors, contributing to optimizing individualized clinical treatments.

For patients with malignant adrenal tumors, we found various clinical factors affecting OS and CSS, especially histological type, tumor size, SEER stage, and surgery. It was reported that pheochromocytoma was accompanied by malignant behaviors in a small number of patients (14); however, the mortality was extremely high. ACC was considered a rare tumor, with a 5-year OS of less than 30% (15, 16). Therefore, the accurate prediction of patient survival would contribute to individualized treatment in clinical therapy. Additionally, tumor size as the clinical variable affecting prognosis also represented tumor burden. The cancer cells had a higher gene mutation probability with a heavier tumor burden (17). For tumors with multiple genetic mutations, mortality was considered higher than typical cancers (18). SEER stage was one kind of tumor grading method from the SEER database, which divided the tumor into localized, regional, distant, and unstage. Several studies reported that SEER stage was valuable in predicting survival for different kinds of cancer (19–21). As for the treatment of malignant adrenal tumors, surgical resection was recognized as the best therapy for the patients (22). Moreover, chemotherapy and radiotherapy were considered adjunctive treatment options after surgical resection. In this study, the nomogram included the above clinical predictive factors for comprehensive prediction of patients’ prognosis, which avoided computational bias.

The C-index, calibration curve, and CIC evaluated the predictive performance of the nomogram in the study. The results indicated the excellent survival predictive accuracy of the nomograms for patients with malignant adrenal tumors. However, there were some limitations in this study. The first limitation would be SEER stage, as the retrospective study cohort had an inevitable statistical bias due to the manual registration process. Additionally, the data retrieved from the SEER database only involved the U.S. population, which could not represent Asian population data. Furthermore, the established nomogram was not applied in clinical practice for OS and CSS prediction. Therefore, multicenter prospective clinical trials will be necessary for subsequent studies to evaluate the nomograms.

## Conclusion

In this study, we identified gender, age, marital status, histological type, tumor size, SEER stage, surgery, and chemotherapy as the prognostic factors affecting patient survival. Meanwhile, we conducted regression analyses for OS and CSS of patients with malignant adrenal tumors and established the nomogram to predict patients’ prognosis based on the statistical results. It was a meaningful attempt to predict prognosis and optimize individualized clinical treatment. This nomogram would assist clinicians in determining the optimal treatment plan for patients.

## Data availability statement

The original contributions presented in the study are included in the article/supplementary material. Further inquiries can be directed to the corresponding authors.

## Ethics statement

We are accountable for all aspects of this study. All procedures performed in this study were in accordance with the Declaration of Helsinki and approved by the Ethics Committee of Shanghai Tenth People’s Hospital. The patients’ confirmation is not applicable for the public available data of clinical samples from SEER database in this study.

## Author contributions

Conception and design: KW, BP, WM, and JW. Administrative support: GW, TZ, and BP. Provision of study materials or patients: TZ, JN, and YG. Collection and assembly of data: HZ, GW, and TZ. Data analysis and interpretation: KW and TZ. Manuscript writing: All authors. Final approval of manuscript: All authors.

## Funding

This work was supported by the National Natural Science Foundation of China (Grant Nos. 81870517 and 32070646), the Shanghai Association for Science and Technology Commission (Grant No. 19140905402), the Science and Technology Innovation Project in Health System of Shanghai Putuo District (Grant No. ptkwws202206), and Incubation Projects of Shanghai Tenth People’s Hospital (Grant No. 04.03.20125).

## Acknowledgments

The authors are grateful for the invaluable support and useful discussions with other members of the Urological Department.

## Conflict of interest

The authors declare that the research was conducted in the absence of any commercial or financial relationships that could be construed as a potential conflict of interest.

## Publisher’s note

All claims expressed in this article are solely those of the authors and do not necessarily represent those of their affiliated organizations, or those of the publisher, the editors and the reviewers. Any product that may be evaluated in this article, or claim that may be made by its manufacturer, is not guaranteed or endorsed by the publisher.
